# Risk of acute exacerbations in chronic obstructive pulmonary disease associated with biomass smoke compared with tobacco smoke

**DOI:** 10.1186/s12890-019-0833-7

**Published:** 2019-03-22

**Authors:** Jaeyoung Cho, Chang-Hoon Lee, Seung-sik Hwang, Ki Uk Kim, Sang Haak Lee, Hye Yun Park, Seoung Ju Park, Kyung Hoon Min, Yeon-Mok Oh, Kwang Ha Yoo, Ki-Suck Jung, on behalf of the KOLD and KOCOSS Investigators

**Affiliations:** 1 Division of Pulmonary and Critical Care Medicine, Department of Internal Medicine, Seoul National University Hospital, 101 Daehak-ro, Jongno-gu, Seoul, 03080 Republic of Korea; 2 Department of Public Health Science, Graduate School of Public Health, Seoul National University, Seoul, Republic of Korea; 3 Department of Internal Medicine, Pusan National University School of Medicine, Busan, Republic of Korea; 4 Division of Pulmonary, Critical Care and Sleep Medicine, Department of Internal Medicine, St. Paul’s Hospital, College of Medicine, The Catholic University of Korea, Seoul, Republic of Korea; 5 Division of Pulmonary and Critical Care Medicine, Department of Medicine, Samsung Medical Center, Sungkyunkwan University School of Medicine, Seoul, Republic of Korea; 6 Department of Internal Medicine, Chonbuk National University Medical School, Jeonju, Republic of Korea; 7 Division of Pulmonary, Allergy and Critical Care Medicine, Department of Internal Medicine, Korea University Guro Hospital, Korea University College of Medicine, Seoul, Republic of Korea; 8 Department of Pulmonary and Critical Care Medicine, Asan Medical Center, University of Ulsan College of Medicine, Seoul, Republic of Korea; 9 Division of Pulmonary and Critical Care Medicine, Department of Internal Medicine, Konkuk University Medical Center, Konkuk University School of Medicine, Seoul, Republic of Korea; 10 Division of Pulmonary, Allergy and Critical Care Medicine, Department of Internal Medicine, Hallym University Sacred Heart Hospital, Hallym University College of Medicine, Anyang, Republic of Korea

**Keywords:** Chronic obstructive pulmonary disease, Biomass smoke, Tobacco smoke, Exacerbation

## Abstract

**Background:**

Risk of exacerbations in chronic obstructive pulmonary disease (COPD) associated with biomass smoke has not been well addressed, although biomass smoke is similar in composition to tobacco smoke.

**Methods:**

To investigate whether the risk of exacerbations in COPD associated with biomass smoke differs from that in COPD associated with tobacco smoke, we recruited patients with COPD from two Korean multicenter prospective cohorts. In a multiple linear regression model, the standardized regression coefficient (β) of biomass smoke exposure ≥25 years was most similar to that (β^′^) of tobacco smoke exposure ≥10 pack-years (β = − 0.13 and β^′^ = − 0.14). We grouped patients with COPD into four categories based on the above cut-offs: Less Tobacco-Less Biomass, Less Tobacco-More Biomass, More Tobacco-Less Biomass, and More Tobacco-More Biomass. The main outcome was the incidence of moderate or severe exacerbations.

**Results:**

Among 1033 patients with COPD, 107 were included in Less Tobacco-Less Biomass (mean age: 67 years, men: 67%), 40 in Less Tobacco-More Biomass (mean age: 70 years, men: 35%), 631 in More Tobacco-Less Biomass (mean age: 68 years, men: 98%), and 255 in More Tobacco-More Biomass (mean age: 69 years, men: 97%). The incidence rates of exacerbations were not significantly different between Less Tobacco-More Biomass and More Tobacco-Less Biomass (adjusted incidence rate ratio, 1.03; 95% confidence interval, 0.56–1.89; *P* = 0.921). No interaction between sex and tobacco and biomass smoke was observed. When propensity score matching with available covariates including age and sex was applied, a similar result was observed.

**Conclusions:**

Patients with COPD associated with biomass smoke and those with COPD associated with tobacco smoke had a similar risk of exacerbations. This suggests that patients with COPD associated with biomass smoke should be treated actively.

**Electronic supplementary material:**

The online version of this article (10.1186/s12890-019-0833-7) contains supplementary material, which is available to authorized users.

## Background

Biomass smoke exposure is an important risk factor for the development of chronic obstructive pulmonary disease (COPD), even though tobacco smoking is the most well-studied COPD risk factor [1–6]. The World Health Organization reported that more than 40% of the world’s population continues to depend on biomass fuels for cooking and heating, and indoor air pollution caused by these activities was responsible for 7.7% of the global mortality in 2012 [7]. Approximately 25% of premature deaths from COPD in low- and middle-income countries are due to exposure to biomass smoke exposure [8].

The clinical characteristics of COPD associated with biomass smoke are different from those associated with tobacco smoke. Patients with COPD associated with biomass smoke are predominantly women, and have worse symptoms and quality of life than do those with COPD associated with tobacco smoke [9, 10]. Recent studies have found phenotypic differences between COPD associated with either biomass or tobacco smoke exposure [10–12]. Biomass smoke exposure is associated with a small airway disease phenotype, whereas tobacco smoke exposure is associated with an emphysema phenotype.

However, only a few longitudinal studies have compared the outcomes between COPD associated with biomass smoke and tobacco smoke [13, 14], and no studies have investigated the risk of exacerbations in patients with COPD exposed to biomass smoke in a prospective study. Exacerbations of COPD are important outcomes in the management of COPD since they negatively impact the health status, hospital admissions and readmissions, disease progression, and mortality [1, 15]. Thus, we aimed to determine whether the risk of exacerbations in COPD associated with biomass smoke differs from that in COPD associated with tobacco smoke. We hypothesized that patients with COPD associated with biomass smoke would have a lower risk of exacerbations than those with COPD associated with tobacco smoke as the rate of FEV_1_ decline is slower in patients whose COPD was associated with biomass smoke than in those whose COPD was associated with tobacco smoke [14].

## Methods

### Patients

We recruited participants from two multicenter prospective cohorts in the Republic of Korea: the Korean Obstructive Lung Disease (KOLD) cohort, which comprises participants from 17 centers recruited since 2005 [16], and the Korean COPD Subgroup Study (KOCOSS; NCT02800499) cohort, which comprises participants from 45 centers recruited since 2011 [17]. Major exclusion criteria of the two cohort studies are patients with respiratory diseases other than obstructive lung disease (e.g., previous pulmonary resection, tuberculosis-destroyed lung, and bronchiectasis). All participants in the cohorts provided written informed consent. Participants were eligible for the current study if they were 40 years or older and had post-bronchodilator forced expiratory volume in 1 s/forced vital capacity (FEV_1_/FVC) less than 0.7. Participants with an unknown exposure history to tobacco and/or biomass fuel smoke were excluded. Participants who were followed up for less than 6 months and those who did not have baseline information were also excluded. The present study was approved by the institutional review board of the Seoul National University Hospital (H-1706-079-859) and was conducted in accordance with the tenets of the Declaration of Helsinki.

### Definition of exposure groups

At a baseline visit, biomass fuel exposure was determined by using the same questions in both cohorts: “Have you ever burned firewood for cooking or heating by yourself for over a year in your lifetime? If yes, how many years have you burned firewood as fuel?” and “Have you ever used coal briquettes for cooking or heating by yourself for over a year in your lifetime? If yes, how many years have you used coal briquettes as fuel?” We defined the exposure years to biomass fuel smoke in this study as the sum of exposure years to firewood and coal briquettes. In the Republic of Korea, coal briquettes had been used as the major source of fuel for cooking and heating since the 1950s; however, they had been replaced by gas and liquid fuels from the early 1990s, and biomass fuel use is now negligible [18]. To find equivalents of exposure to tobacco and biomass smoke, we applied multiple linear regression in the 1031 cohort participants (mean age, 69 years; 934 men; 55 non-COPD) who were not currently exposed to tobacco and biomass smoke.

$$ \mathrm{E}\left(\mathrm{Y}\right)=\Big\{{\displaystyle \begin{array}{l}{\mathrm{B}}_0+{\mathrm{B}}_1{\mathrm{X}}_1+{\mathrm{B}}_2{\mathrm{X}}_2+{\mathrm{B}}_3{\mathrm{X}}_3+{\mathrm{B}}_4{\mathrm{X}}_4+\upvarepsilon, \mathrm{if}\ {\mathrm{X}}_5=0\\ {}{\mathrm{B}}_0^{\prime }+{\mathrm{B}}_1^{\prime }{\mathrm{X}}_1+{\mathrm{B}}_2^{\prime }{\mathrm{X}}_2+{\mathrm{B}}_3^{\prime }{\mathrm{X}}_3+{\mathrm{B}}_5^{\prime }{\mathrm{X}}_5+{\upvarepsilon}^{\prime },\mathrm{if}\ {\mathrm{X}}_4=0\end{array}}\operatorname{} $$

In the above equation, Y is post-bronchodilator FEV_1_/FVC (%) at a baseline visit; X_1_ is age (0 if age < 60 years and 1 if age ≥ 60 years); X_2_ is sex (0 if male and 1 if female); X_3_ is height (cm), X_4_ is tobacco smoke (0 if < *m* pack-years and 1 if ≥ *m* pack-years, where *m* is an integer); X_5_ is biomass smoke (0 if < *n* years and 1 if ≥ *n* years, where *n* is an integer); B_0_, ${\mathrm{B}}_0^{\prime }$ are the intercept terms; B_1_, ${\mathrm{B}}_1^{\prime }$ to B_4_, ${\mathrm{B}}_5^{\prime }$ are regression coefficients; and ε, ε^′^ are the random error terms. The standardized regression coefficient [19] of B_4_ (β) and that of ${\mathrm{B}}_5^{\prime }$ (β^′^) were most similar when *m* was 10 and *n* was 25 (β = − 0.13 and β^′^ = − 0.14) (Table 1). Therefore, the tobacco group (More Tobacco) was defined as one with ≥10 pack-years of tobacco smoke, and the biomass group (More Biomass) was defined as one with ≥25 exposure years to biomass smoke. We grouped patients with COPD into four categories based on the above cut-offs: Less Tobacco-Less Biomass, Less Tobrefacco-More Biomass, More Tobacco-Less Biomass, and More Tobacco-More Biomass.

**Table 1:** The equivalents of pack-years of tobacco smoke and exposure years to biomass smoke^*^

		If biomass smoke < *n* years				If tobacco smoke < *m* pack-years			
Tobacco smoke (*m*), pack-years	Exposure to biomass smoke (*n*), years	B_4_	SE	β	*P* value	${\mathrm{B}}_5^{\prime }$	SE	β^′^	*P* value
10	10	−4.22	1.79	−0.12	0.019	−0.15	1.87	−0.01	0.937
	15	−3.48	1.48	−0.10	0.019	0.37	1.83	0.02	0.840
	20	−3.51	1.44	−0.11	0.015	−0.14	1.83	−0.01	0.941
	25	−4.20	1.32	−0.13	0.002	−3.83	1.92	−0.14	0.048
	30	−4.12	1.30	−0.13	0.002	−2.24	2.00	−0.08	0.263
20	10	−4.36	1.43	−0.15	0.003	0.51	1.59	0.02	0.749
	15	−3.60	1.24	−0.13	0.004	0.74	1.58	0.03	0.639
	20	−3.62	1.21	−0.13	0.003	0.38	1.60	0.02	0.811
	25	−4.39	1.11	−0.15	< 0.001	−3.25	1.68	−0.12	0.054
	30	−4.01	1.09	−0.14	< 0.001	−1.38	1.73	−0.05	0.424

^*^Multiple linear regression adjusting for age (< 60 vs. ≥60 years), sex, and height (cm). The standardized regression coefficient of B_4_ (β) and that of ${\mathrm{B}}_5^{\prime }$ (β^′^) were most similar when *m* was 10 and *n* was 25 (β = − 0.13 and β^′^ = − 0.14)

*B*_4_ unstandardized regression coefficient for tobacco smoke, ${B}_5^{\prime }$ unstandardized regression coefficient for biomass smoke, *β* standardized regression coefficient of B_4_, *β*^′^ standardized regression coefficient of ${\mathrm{B}}_5^{\prime }$, *SE* standard error

### Measurement of variables and outcomes

All participants underwent detailed interviews by study physicians or trained nurses covering an exposure history to tobacco and biomass smoke; a history of exacerbations during the previous year; and symptom scores, including those for the modified Medical Research Council (mMRC) Questionnaire [20], St. George’s respiratory questionnaire for COPD (SGRQ-C) [21], and COPD assessment test (CAT) [22]. Participants underwent pre- and post-bronchodilator spirometry at baseline. After a baseline visit, participants were followed up every 3 months (the KOLD cohort) or 6 months (the KOCOSS cohort). The use of drugs, including long-acting muscarinic antagonists, long-acting β-agonists, and inhaled corticosteroids at enrollment and the medication possession ratios of those drugs during the follow-up period were recorded.

The main outcome was the incidence of moderate or severe exacerbations. An exacerbation was defined as moderate when any worsening of respiratory symptoms led to treatment with systemic corticosteroids, antibiotics, or both, and severe if it led to hospital admission or emergency department visits [15, 23].

### Statistical analysis

Clinical characteristics were compared using independent samples *t*-tests or one-way analysis of variance for continuous variables. For categorical variables, comparisons were made using either χ^2^ tests or Fisher’s exact tests. The incidence rates of moderate or severe exacerbations were compared in the four exposure groups by using negative binomial regression models. The time to the first moderate or severe exacerbation was analyzed using the Kaplan–Meier method. Thereafter, we repeated the analyses after propensity score matching. Multivariable logistic regression was used to compute the propensity score for the Less Tobacco-More Biomass group by using available covariates. The Less Tobacco-More Biomass group was matched (1:3 ratio) with the More Tobacco-Less Biomass group by using the nearest neighbor method within a caliper of 0.3 of the propensity score. We also performed sensitivity analysis with four groups of patients (never exposed to tobacco or biomass smoke, exposed to biomass smoke only, exposed to tobacco smoke only, and exposed to both biomass and tobacco smoke). *P* values less than 0.05 were considered significant. Statistical analyses were performed using Stata statistical software (Version 14.2; StataCorp, College Station, TX).

## Results

**Fig. 1:**
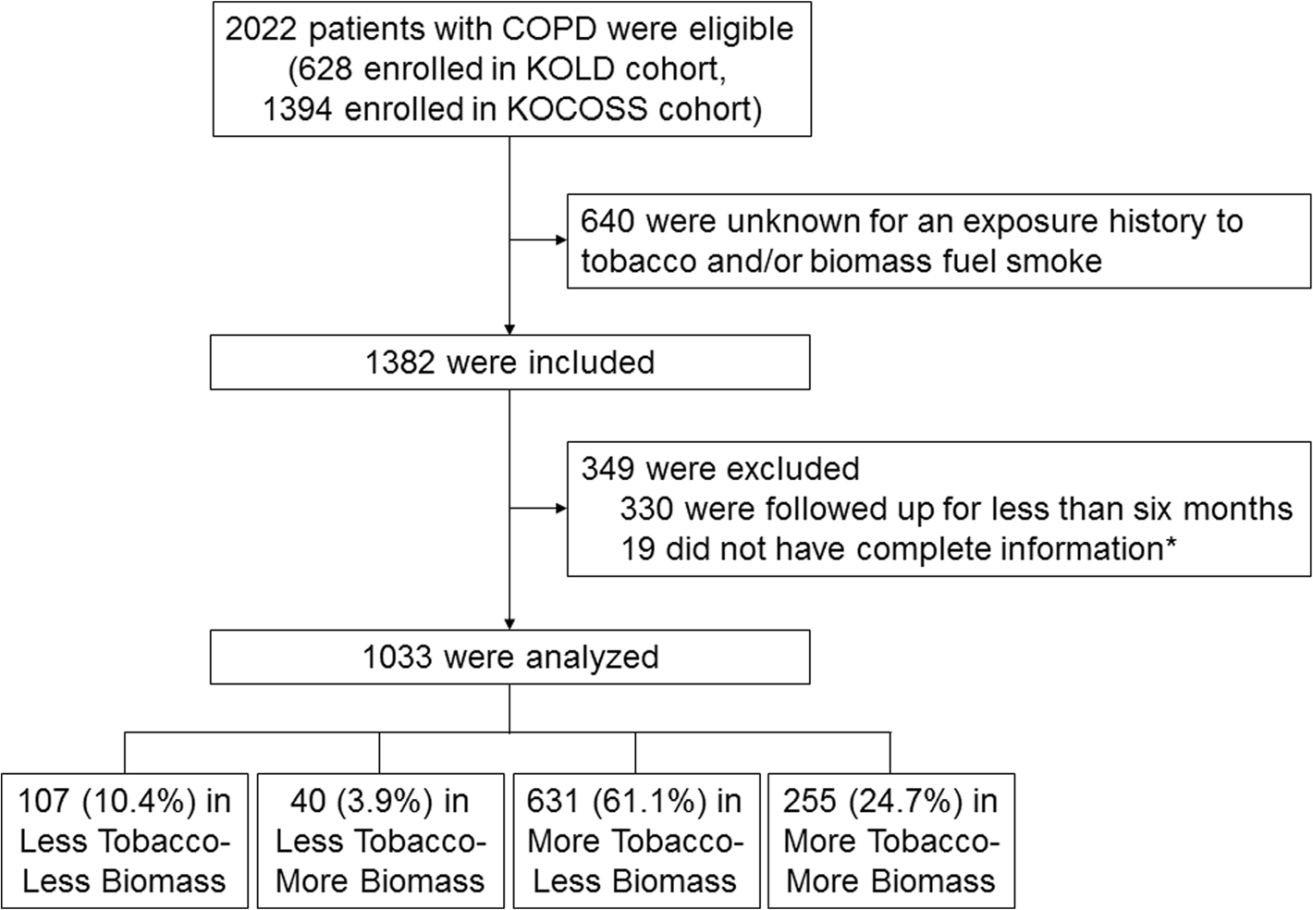
Flowchart illustrating patient selection. ^*^Body mass index, % predicted post-bronchodilator forced expiratory volume in 1 s (FEV_1_), exacerbation history during the previous year, the modified Medical Research Council dyspnea score, and the St. George’s respiratory questionnaire for COPD score at baseline *COPD* chronic obstructive pulmonary disease, *KOCOSS* Korean COPD Subgroup Study, *KOLD* Korean Obstructive Lung Disease.

Among the 1033 patients with COPD who were followed up for a mean duration of 3.0 years, 107 (10.4%) were included in the Less Tobacco-Less Biomass group, 40 (3.9%) in the Less Tobacco-More Biomass group, 631 (61.1%) in the More Tobacco-Less Biomass group, and 255 (24.7%) in the More Tobacco-More Biomass group (Fig. 1). Characteristics of the study patients according to exposure are presented in Table 2. Patients in the Less Tobacco-More Biomass group were more likely to be women (65.0 vs. 2.2%; *P* < 0.001) and older (69.9 ± 6.5 vs. 67.5 ± 7.6 years; *P* = 0.061) than those in the More Tobacco-Less Biomass group (see Additional file 1: Table S1). Although the mMRC dyspnea scores were similar in the four groups, comprehensive symptom assessment questionnaires showed that the Less Tobacco-More Biomass group had significantly more symptoms. Post-bronchodilator FEV_1_% predicted was not significantly different across the groups (Table 2).

**Table 2:** Baseline characteristics of the study patients^*^

	Less Tobacco-Less Biomass (*n* = 107)	Less Tobacco-More Biomass (*n* = 40)	More Tobacco-Less Biomass (*n* = 631)	More Tobacco-More Biomass (*n* = 255)	*P* value
Age, years	67.1 ± 9.3	69.9 ± 6.5	67.5 ± 7.6	69.2 ± 7.1	0.007
Age					0.004
< 60 years	23 (21.5)	2 (5.0)	99 (15.7)	24 (9.4)	
≥ 60 years	84 (78.5)	38 (95.0)	532 (84.3)	231 (90.6)	
Sex					< 0.001
Male	72 (67.3)	14 (35.0)	617 (97.8)	248 (97.3)	
Female	35 (32.7)	26 (65.0)	14 (2.2)	7 (2.8)	
BMI, kg/m^2^	23.7 ± 3.3	23.7 ± 3.3	23.0 ± 3.1	22.5 ± 3.4	0.005
Tobacco smoking					< 0.001
Never-smoker	70 (65.4)	31 (77.5)	0 (0.0)	0 (0.0)	
Former smoker	32 (29.9)	9 (22.5)	447 (70.8)	165 (64.7)	
Current smoker	5 (4.7)	0 (0.0)	184 (29.2)	90 (35.3)	
Tobacco smoke, pack-years	1.7 ± 2.9	0.8 ± 2.1	45.8 ± 24.9	47.1 ± 25.4	< 0.001
Biomass smoke, years	8.9 ± 7.9	35.5 ± 8.3	5.9 ± 7.5	37.9 ± 11.2	< 0.001
Exacerbation during the previous year					0.164
No	79 (73.8)	25 (62.5)	485 (76.9)	199 (78.0)	
Yes	28 (26.2)	15 (37.5)	146 (23.1)	56 (22.0)	
mMRC dyspnea score	1.4 ± 0.9	1.7 ± 0.9	1.5 ± 0.9	1.6 ± 0.9	0.083
mMRC dyspnea score					0.455
< 2	63 (58.9)	19 (47.5)	367 (58.2)	139 (54.5)	
≥ 2	44 (41.1)	21 (52.5)	264 (41.8)	116 (45.5)	
SGRQ-C total score	32.8 ± 18.3	44.4 ± 21.7	32.1 ± 18.1	36.8 ± 18.3	< 0.001
SGRQ-C total score					0.001
< 25	44 (41.1)	9 (22.5)	269 (42.6)	77 (30.2)	
≥ 25	63 (58.9)	31 (77.5)	362 (57.4)	178 (69.8)	
CAT score (*n* = 780)	13.9 ± 7.9	18.7 ± 10.0	14.0 ± 7.8	16.2 ± 8.0	< 0.001
CAT score					< 0.001
< 10	30 (28.0)	6 (15.0)	156 (24.7)	30 (11.8)	
≥ 10	63 (58.9)	23 (57.5)	339 (53.7)	133 (52.2)	
Unknown	14 (13.1)	11 (27.5)	136 (21.6)	92 (36.1)	
Post-bronchodilator FEV_1_, % predicted	59.3 ± 18.6	63.4 ± 24.2	60.3 ± 17.7	60.0 ± 19.4	0.685
Post-bronchodilator FVC, % predicted	80.8 ± 17.1	84.3 ± 20.7	88.6 ± 17.2	85.5 ± 18.9	< 0.001
Post-bronchodilator FEV_1_/FVC, %	53.4 ± 10.3	53.1 ± 12.5	48.3 ± 11.3	49.2 ± 11.3	< 0.001
Bronchodilator response (FEV_1_, %)	6.4 ± 11.4	8.0 ± 13.7	8.5 ± 10.9	8.6 ± 9.7	0.270
Blood eosinophil, % (*n* = 868)	3.6 ± 4.4	2.4 ± 1.7	3.6 ± 3.4	3.7 ± 4.0	0.196
Blood eosinophil					< 0.001
≤ 5%	68 (63.6)	35 (87.5)	409 (64.8)	180 (70.6)	
> 5%	19 (17.8)	2 (5.0)	101 (16.0)	54 (21.2)	
Unknown	20 (18.7)	3 (7.5)	121 (19.2)	21 (8.2)	
Use of LAMA at enrollment	57 (53.3)	16 (40.0)	329 (52.1)	113 (44.3)	0.090
Use of LABA at enrollment	54 (50.5)	26 (65.0)	329 (52.1)	144 (56.5)	0.273
Use of ICS at enrollment	38 (35.5)	22 (55.0)	263 (41.7)	114 (44.7)	0.147

^*^Data are presented as mean ± SD or No. (%)

*BMI* Body mass index, *CAT* COPD Assessment Test, *COPD* chronic obstructive pulmonary disease, *FEV*_*1*_ forced expiratory volume in 1 s, *FVC* forced vital capacity, *ICS* inhaled corticosteroid, *LABA* long-acting β-agonist, *LAMA* long-acting muscarinic antagonist, *mMRC* modified Medical Research Council, *SGRQ-C* St. George’s Respiratory Questionnaire for COPD

**Table 3:** Adjusted incidence rates of moderate or severe exacerbations by exposure groups

	Adjusted incidence rate^*^ (95% CI)	Adjusted incidence rate ratio^*^ (95% CI)	*P* value
More Tobacco-Less Biomass	0.70 (0.60–0.79)	1	
Less Tobacco-Less Biomass	0.51 (0.33–0.68)	0.73 (0.50–1.05)	0.092
Less Tobacco-More Biomass	0.72 (0.31–1.13)	1.03 (0.56–1.89)	0.921
More Tobacco-More Biomass	0.67 (0.54–0.80)	0.96 (0.76–1.20)	0.698

^*^Adjusted for age, sex, body mass index, St. George’s Respiratory Questionnaire for COPD (SGRQ-C) total score (< 25 vs. ≥25), exacerbation history during the previous year (yes vs. no), and post-bronchodilator forced expiratory volume in 1 s (FEV_1_)% predicted

*CI* confidence interval

**Fig. 2:**
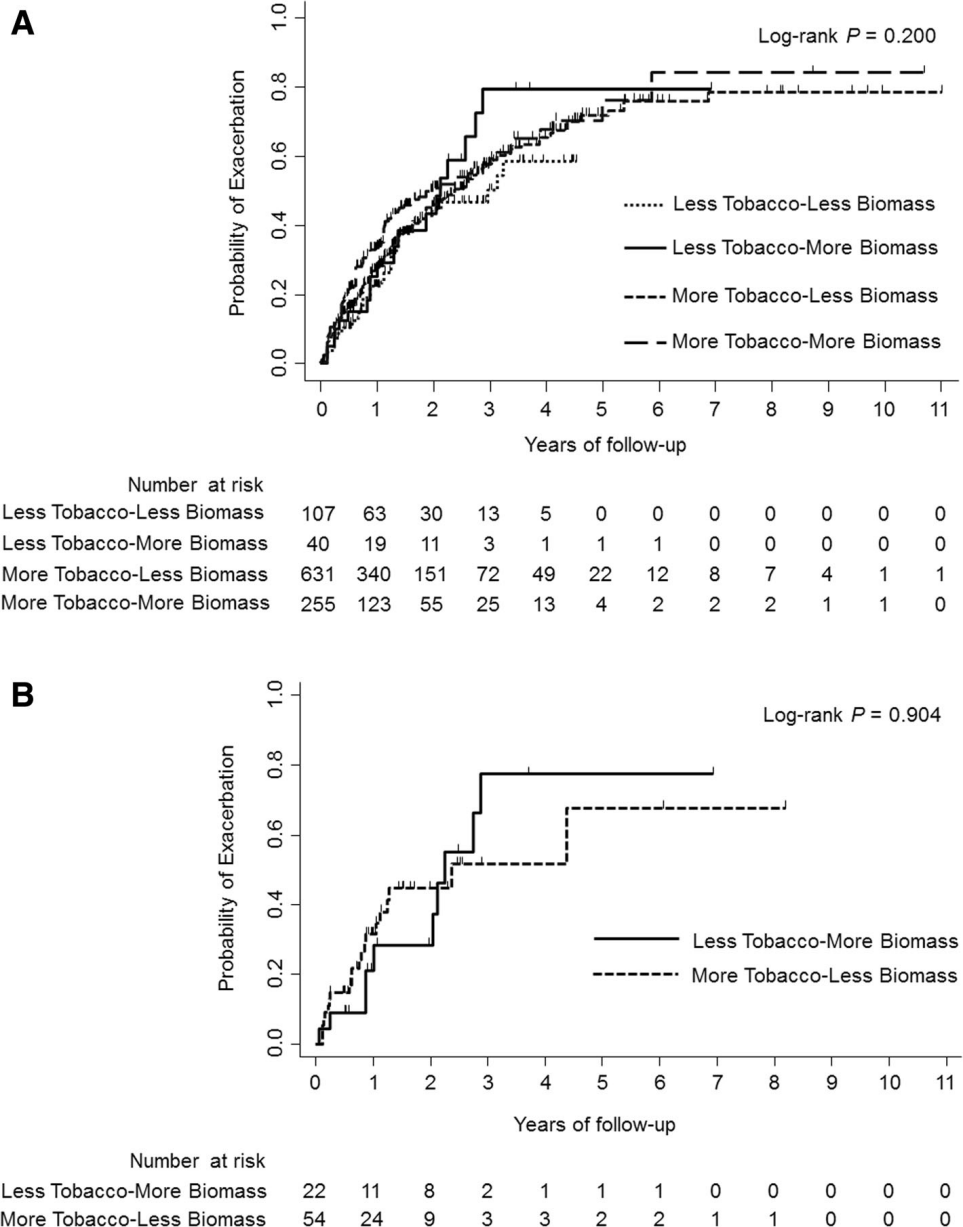
Kaplan–Meier curves illustrating the time to the first moderate or severe exacerbation in four exposure groups (**a**) and in two exposure groups after propensity score matching (**b**)

When adjusted for age, sex, body mass index, SGRQ-C total score (< 25 vs. ≥25), exacerbation history during the previous year (yes vs. no), and post-bronchodilator FEV_1_% predicted, the incidence rates of moderate or severe exacerbations did not differ by groups (Table 3). The adjusted incidence rates were 0.72 (95% confidence interval [CI], 0.31–1.13) in the Less Tobacco-More Biomass group and 0.70 (95% CI, 0.60–0.79) in the More Tobacco-Less Biomass group, resulting in an incidence rate ratio of 1.03 (95% CI, 0.56–1.89; *P* = 0.921). No interaction between sex and tobacco smoke (< 10 vs. ≥10 pack-years) and biomass smoke (< 25 vs. ≥25 years) was observed (data not shown). In addition, the time to the first moderate or severe exacerbation was not significantly different between the four groups (log-rank *P* = 0.200) (Fig. 2a).

After propensity score matching, each of 16 participants in the Less Tobacco-More Biomass group was matched with three controls in the More Tobacco-Less Biomass group, whereas each of six participants in the Less Tobacco-More Biomass group was matched with a control in the More Tobacco-Less Biomass group. The clinical characteristics of these matched groups were comparable (see Additional file 1: Figure S1 and Table S1). The incidence rates of exacerbations were not significantly different between the groups (incidence rate ratio, 1.18; 95% CI, 0.53–2.59; *P* = 0.687) (Table 4). A similar result was observed in the time to the first exacerbation (log-rank *P* = 0.904) (Fig. 2b).

**Table 4:** Incidence rates of moderate or severe exacerbations in the propensity score-matched cohort

		Incidence rate (95% CI)	Incidence rate ratio (95% CI)	*P* value
Model 1^*^	More Tobacco-Less Biomass	0.58 (0.33–0.83)	1	
	Less Tobacco-More Biomass	0.68 (0.23–1.13)	1.18 (0.53–2.59)	0.687
Model 2^†^	More Tobacco-Less Biomass	0.58 (0.32–0.83)	1	
	Less Tobacco-More Biomass	0.69 (0.21–1.17)	1.19 (0.51–2.78)	0.685
Model 3^‡^	More Tobacco-Less Biomass	0.56 (0.32–0.79)	1	
	Less Tobacco-More Biomass	0.65 (0.22–1.07)	1.16 (0.52–2.58)	0.721

^*^Model 1: unadjusted

^†^Model 2: adjusted for sex

^‡^Model 3: adjusted for sex, and medication possession ratios of long-acting muscarinic antagonists, long-acting β-agonists, and inhaled corticosteroids during the follow-up period as continuous variables

*CI* confidence interval

We performed sensitivity analysis with 22 patients who were never exposed to tobacco or biomass smoke and 79 exposed to biomass smoke only and 322 exposed to tobacco smoke only and 610 exposed to both biomass and tobacco smoke. We found no difference in the rates of exacerbations between COPD associated with biomass smoke and that associated with tobacco smoke (see Additional file 1: Table S2). We also applied a propensity score-matched analysis. Forty participants exposed to biomass smoke only were matched with 40 participants exposed to tobacco smoke only. The incidence rates of exacerbations were not significantly different between the groups (incidence rate ratio, 1.05; 95% CI, 0.55–1.98; *P* = 0.890; see Additional file 1: Table S3).

## Discussion

The present study investigated whether the risk of exacerbations differs by exposure to biomass or tobacco smoke. We found no difference in the rates of exacerbations between COPD associated with biomass smoke and that associated with tobacco smoke, even in an analysis with propensity score matching.

When an individual with significant exposures to risk factors, such as tobacco smoke, indoor/outdoor air pollution, and occupational dusts and chemicals, presents appropriate symptoms, a diagnosis of COPD is made on the basis of spirometry, which confirms the presence of persistent airflow limitation [1]. Tobacco smoke continues to be recognized as the most commonly encountered risk factor for COPD. In most clinical studies including large trials, a history of tobacco smoke exposure has been accepted as a major inclusion criterion to define COPD [24–27]. The pathogenesis and pathophysiology of COPD caused by tobacco smoke have been well-studied, and in vivo and in vitro models of cigarette smoke-induced COPD have been widely used [28]. Biomass smoke exposure is now being identified as an important risk factor for persistent airflow limitation. However, translational and clinical research on COPD associated with biomass smoke has been limited, especially from the perspective of comparing exposure to biomass smoke and to tobacco smoke [29]. In a Mexican cohort, the rate of FEV_1_ decline was slower and more homogeneous in patients whose COPD was associated with biomass smoke than in those whose COPD was associated with tobacco smoke [14]. In the same cohort, no differences were found in survival between the two groups after adjusting for confounders [13]. However, the risk of exacerbations, another important COPD outcome, in the patients exposed to biomass smoke has not been well addressed as a few studies investigated it retrospectively [13, 30].

To our knowledge, ours is the first prospective study comparing the risk of exacerbations between COPD associated with biomass smoke and that associated with tobacco smoke. We recruited 1033 patients with COPD and grouped them into four categories based on the presence of risk factors, biomass and/or tobacco smoke. Patients with COPD exposed to biomass smoke were predominantly women and older, and presented more symptoms even with a similar degree of airflow limitation than did those exposed to tobacco smoke. After adjusting for confounding factors, the rates of moderate or severe exacerbations did not differ by exposure groups. When propensity score matching was applied to compare participants with either biomass or tobacco smoke, a similar result was observed. Biomass and tobacco smoke shares common harmful components [31], and a recent experimental study showed that biomass fuel smoke activated similar pathogenic processes observed in cigarette smoke exposure in both human airway epithelial cells and mice [32]. A considerable amount of evidence, from in vivo and in vitro studies, shows biomass smoke enhances lung inflammation and impairs pulmonary anti-microbial defense, which could lead to exacerbations [31].

Our finding is consistent with that of previous retrospective analyses reporting no differences in exacerbations between patients with COPD associated with tobacco or biomass smoke [13, 30]. On combining our finding with that of the previous cohort study, which demonstrated similar mortality between patients with COPD exposed to the two different smokes [13], we found that clinical and radiological differences between COPD associated with biomass and tobacco smoke may not lead to significant differences in clinical outcomes. Therefore, clinicians should suspect a diagnosis of COPD in any patient with a history of exposure to biomass fuel smoke who has dyspnea, chronic cough, or sputum production, and any patient with COPD associated with biomass smoke should be treated actively.

As the cumulative amounts of exposure to biomass smoke, which are comparable to those of exposure to tobacco smoke, are unknown, the group exposed to biomass smoke has been defined using arbitrary cut-off values of exposure duration in years, especially when an individual’s exposure intensity to biomass smoke is unavailable [30, 33]. Cumulative exposure to biomass smoke expressed as hour-years, which is the product of the number of years cooking with biomass fuels multiplied by the average number of hours spent daily in the kitchen [34], was unavailable in our cohort. Instead, we used the standardized regression coefficient to determine equivalents of the exposure duration (years) to biomass smoke and pack-years of tobacco smoke. The exposure duration to biomass smoke was defined as the sum of exposure years to firewood and coal briquettes. We applied multiple linear regression predicting values of the dependent variable, post-bronchodilator FEV_1_/FVC (%) at a baseline visit, in the 1031 cohort participants including non-COPD in a cross-sectional design. Upon repeated modeling with variable values, we found that the standardized regression coefficients for each exposure were most similar when the exposure duration to biomass smoke was 25 years and a history of tobacco smoke was 10 pack-years (Table 1). However, there are several limitations in these estimates. First, we thought that a multiple linear regression model predicting values of the dependent variable, not post-bronchodilator FEV_1_/FVC (%) but diagnosis of COPD, would be ideal for determining comparable cut-off values of exposures. While there were only 5% participants without COPD in the KOLD and KOCOSS cohorts, other prospective cohorts utilizing the same questionnaire on a history of exposure to biomass smoke were not available in Korea. Second, our regression model using the spirometric value as a dependent variable is still limited by the small number of participants without COPD. Third, questions assessing biomass exposure were focused on cooking or heating. However, it is possible that a responder was not a cook, but still had been exposed to biomass smoke. Fourth, the exposure duration to biomass smoke was defined as the simple sum of exposure years to firewood and coal briquettes without weights although the risk of development of COPD was greater for wood burners than for coal users [3]. Fifth, exposure to tobacco and/or biomass smoke under the cut-off values may cause COPD. Sixth, exposure data on other risk factors for COPD, such as indoor/outdoor air pollution and occupational dusts, were lacking in our study.

Sex selection bias is a commonly encountered issue in COPD research associated with biomass smoke [11]. The present study also has the limitation as patients with COPD associated with biomass smoke were predominantly women. To mitigate the bias, we applied a propensity score-matched analysis with available covariates including sex. This approach resulted in the findings consistent with the overall results on COPD exacerbation rates. In addition, no interaction was observed between sex and tobacco and biomass smoke both before and after matching. However, although controversies still arise, women can be more susceptible of the effects of tobacco and biomass smoke then men, leading to more severe disease for the equivalent quantity of smoke [1, 35].

The current study has several limitations, such as its observational design and the small number of patients with COPD associated with biomass smoke. There was a possibility of recall bias as the data regarding exposure to biomass and tobacco smoke were collected at the time of or after the diagnosis of COPD. Although we included participants aged 40 years or older, COPD associated with biomass smoke could be prevalent in younger adults [36].

## Conclusions

In conclusion, this study shows that patients with COPD associated with biomass smoke and those with COPD associated with tobacco smoke have a similar risk of exacerbations. This suggests that any patient with COPD associated with biomass smoke should be treated actively. In addition, management strategies should not be confined to pharmacologic treatment, and should be complemented by interventions, such as improvement of kitchen ventilation, introduction of non-polluting cooking stoves, and use of clean fuels, to reduce exposure to smoke from biomass fuels.

## Additional file

Additional file 1: Online supplement (DOC). **Figure S1.** Histograms showing propensity score distribution. **Table S1.** Baseline characteristics of the study patients before and after propensity score matching. **Table S2.** Adjusted incidence rates of moderate or severe exacerbations by exposure groups (sensitivity analysis). **Table S3.** Incidence rates of moderate or severe exacerbations in the propensity score-matched cohort (sensitivity analysis) (DOC 117 kb)

## Abbreviations

CAT: COPD assessment test
COPD: chronic obstructive pulmonary disease
KOCOSS: Korean COPD Subgroup Study
KOLD: Korean Obstructive Lung Disease
mMRC: modified Medical Research Council
SGRQ-C: St. George’s respiratory questionnaire for COPD
