# Supplementary material for: Risk of acute exacerbations in chronic obstructive pulmonary disease associated with biomass smoke compared with tobacco smoke
Source: BMC Pulm Med. 2019 Mar 22;19:68. doi: 10.1186/s12890-019-0833-7 (PMC6429752; doi:10.1186/s12890-019-0833-7)
Supplement: Supplementary file 1 — Online supplement (DOC). Figure S1. Histograms showing propensity score distribution. Table S1. Baseline characteristics of the study patients before and after propensity score matching. Table S2. Adjusted incidence rates of moderate or severe exacerbations by exposure groups (sensitivity analysis). Table S3. Incidence rates of moderate or severe exacerbations in the propensity score-matched cohort (sensitivity analysis) (DOC 117 kb) [file 12890_2019_833_MOESM1_ESM.doc]

**Additional file 1**

**Risk of Acute Exacerbations in Chronic Obstructive Pulmonary Disease Associated with Biomass Smoke Compared with Tobacco Smoke**

**Figure S1***-* Histograms showing propensity score distribution. (TIFF)


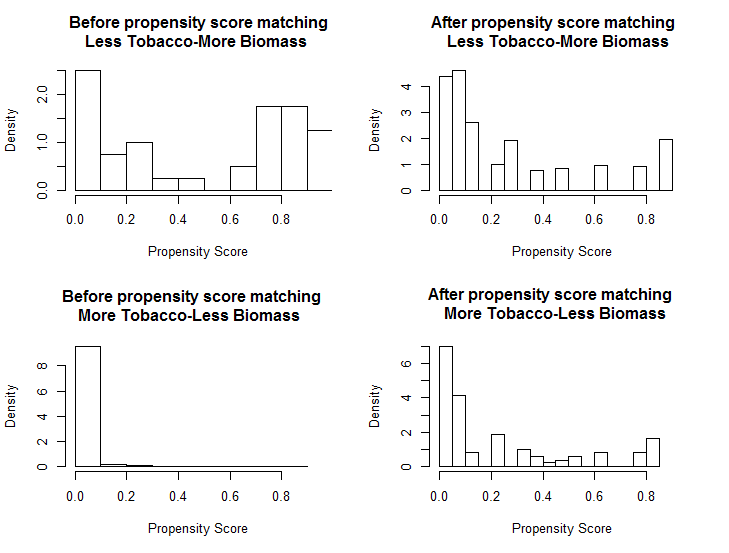


**Table S1-**Baseline characteristics of the study patients before and after propensity score matching^*^

| Characteristic | Before propensity score matching | | | After propensity score matching^†^ | | |
| --- | --- | --- | --- | --- | --- | --- |
|  | Less Tobacco-More Biomass  (n = 40) | More Tobacco-Less Biomass  (n = 631) | *P* Value | Less Tobacco-More Biomass  (n = 22) | More Tobacco-Less Biomass  (n = 54) | *P* value |
| Age, years | 69.9 ± 6.5 | 67.5 ± 7.6 | 0.061 | 69.1 ± 7.1 | 69.7 ± 7.2 | 0.756 |
| Age |  |  | 0.067 |  |  | >0.999 |
| <60 years | 2 (5.0) | 99 (15.7) |  | 2 (9.1) | 5 (9.3) |  |
| ≥60 years | 38 (95.0) | 532 (84.3) |  | 20 (90.9) | 49 (90.7) |  |
| Sex |  |  | <0.001 |  |  | 0.065 |
| Male | 14 (35.0) | 617 (97.8) |  | 13 (59.1) | 43 (79.6) |  |
| Female | 26 (65.0) | 14 (2.2) |  | 9 (40.9) | 11 (20.4) |  |
| BMI, kg/m^2^ | 23.7 ± 3.3 | 23.0 ± 3.1 | 0.206 | 22.7 ± 2.4 | 22.8 ± 3.3 | 0.899 |
| Tobacco smoking |  |  | <0.001 |  |  | <0.001 |
| Never-smoker | 31 (77.5) | 0 (0.0) |  | 15 (68.2) | 0 (0.0) |  |
| Former smoker | 9 (22.5) | 447 (70.8) |  | 7 (31.8) | 34 (63.0) |  |
| Current smoker | 0 (0.0) | 184 (29.2) |  | 0 (0.0) | 20 (37.0) |  |
| Tobacco smoke, pack-years | 0.8 ± 2.1 | 45.8 ± 24.9 | <0.001 | 1.5 ± 2.8 | 39.0 ± 22.0 | <0.001 |
| Biomass smoke, years | 35.5 ± 8.3 | 5.9 ± 7.5 | <0.001 | 35.2 ± 6.1 | 6.7 ± 8.0 | <0.001 |
| Exacerbations during the previous year |  |  | 0.039 |  |  | 0.837 |
| No | 25 (62.5) | 485 (76.9) |  | 16 (72.7) | 38 (70.4) |  |
| Yes | 15 (37.5) | 146 (23.1) |  | 6 (27.3) | 16 (29.6) |  |
| mMRC dyspnea score | 1.7 ± 0.9 | 1.5 ± 0.9 | 0.120 | 1.5 ± 0.7 | 1.6 ± 0.9 | 0.832 |
| mMRC dyspnea score |  |  | 0.186 |  |  | 0.556 |
| <2 | 19 (47.5) | 367 (58.2) |  | 11 (50.0) | 31 (57.4) |  |
| ≥2 | 21 (52.5) | 264 (41.8) |  | 11 (50.0) | 23 (42.6) |  |
| SGRQ-C total score | 44.4 ± 21.7 | 32.1 ± 18.1 | <0.001 | 39.0 ± 23.5 | 33.4 ± 17.0 | 0.248 |
| SGRQ-C total score |  |  | 0.012 |  |  | 0.956 |
| <25 | 9 (22.5) | 269 (42.6) |  | 8 (36.4) | 20 (37.0) |  |
| ≥25 | 31 (77.5) | 362 (57.4) |  | 14 (63.6) | 34 (63.0) |  |
| CAT score^‡^ | 18.7 ± 10.0 | 14.0 ± 7.8 | 0.021 | 18.9 ± 10.4 | 14.2 ± 7.4 | 0.053 |
| CAT score |  |  | 0.336 |  |  | 0.912 |
| <10 | 6 (15.0) | 156 (24.7) |  | 4 (18.2) | 11 (20.4) |  |
| ≥10 | 23 (57.5) | 339 (53.7) |  | 13 (59.1) | 29 (53.7) |  |
| Unknown | 11 (27.5) | 136 (21.6) |  | 5 (22.7) | 14 (25.9) |  |
| Post-bronchodilator FEV_1_, % predicted | 63.4 ± 24.2 | 60.3 ± 17.7 | 0.430 | 60.2 ± 23.4 | 57.4 ± 18.8 | 0.588 |
| Post-bronchodilator FVC, % predicted | 84.3 ± 20.7 | 88.6 ± 17.2 | 0.124 | 81.8 ± 21.4 | 81.8 ± 19.7 | 0.994 |
| Post-bronchodilator FEV_1_/FVC, % | 53.1 ± 12.5 | 48.3 ± 11.3 | 0.009 | 52.3 ± 12.6 | 48.5 ± 11.7 | 0.221 |
| Bronchodilator response (FEV_1_, %) | 8.0 ± 13.7 | 8.5 ± 10.9 | 0.824 | 7.8 ± 9.2 | 9.2 ± 14.2 | 0.609 |
| Blood eosinophil, %^§^ | 2.4 ± 1.7 | 3.6 ± 3.4 | <0.001 | 2.2 ± 1.5 | 2.9 ± 2.5 | 0.189 |
| Blood eosinophil |  |  | 0.013 |  |  | 0.882 |
| ≤5% | 35 (87.5) | 409 (64.8) |  | 18 (81.8) | 45 (83.3) |  |
| >5% | 2 (5.0) | 101 (16.0) |  | 1 (4.6) | 4 (7.4) |  |
| Unknown | 3 (7.5) | 121 (19.2) |  | 3 (13.6) | 5 (9.3) |  |
| Use of LAMA at enrollment | 16 (40.0) | 329 (52.1) | 0.136 | 11 (50.0) | 26 (48.2) | 0.884 |
| Use of LABA at enrollment | 26 (65.0) | 329 (52.1) | 0.114 | 13 (59.1) | 29 (53.7) | 0.668 |
| Use of ICS at enrollment | 22 (55.0) | 263 (41.7) | 0.098 | 10 (45.5) | 20 (37.0) | 0.496 |

^*^Data are presented as mean ± SD or No. (%).

^†^Each of the 16 subjects in the Less Tobacco-More Biomass group was matched with three controls in the More Tobacco-Less Biomass group, whereas each of the six in the Less Tobacco-More Biomass group was matched with a control in the More Tobacco-Less Biomass group.

^‡^Data are for 524 patients before propensity score matching, and 57 after propensity score matching.

^§^Data are for 547 patients before propensity score matching, and 68 after propensity score matching.

*BMI* body mass index, *CAT* COPD Assessment Test, *COPD* chronic obstructive pulmonary disease, *FEV_1_* forced expiratory volume in 1 s, *FVC* forced vital capacity, *ICS* inhaled corticosteroid, *LABA* long-acting β-agonist, *LAMA* long-acting muscarinic antagonist, *mMRC* modified Medical Research Council, *SGRQ-C* St. George’s Respiratory Questionnaire for COPD

**Table S2-** Adjusted incidence rates of moderate or severe exacerbations by exposure groups (sensitivity analysis)

|  | Adjusted incidence rate^*^  (95% CI) | Adjusted incidence rate ratio^*^  (95% CI) | *P* value |
| --- | --- | --- | --- |
| Tobacco smoke only | 0.73 (0.59–0.86) | 1 |  |
| Never-exposed to tobacco or biomass smoke | 0.16 (0.01–0.31) | 0.22 (0.09–0.56) | 0.001 |
| Biomass smoke only | 0.60 (0.34–0.86) | 0.83 (0.51–1.33) | 0.437 |
| Biomass and tobacco smoke | 0.67 (0.58–0.76) | 0.92 (0.74–1.51) | 0.477 |

^*^Adjusted for age, sex, body mass index, St. George’s Respiratory Questionnaire for COPD (SGRQ-C) total score (<25 vs. ≥25), exacerbation history during the previous year (yes vs. no), and post-bronchodilator forced expiratory volume in 1 s (FEV_1_)% predicted.

**Table S3-** Incidence rates of moderate or severe exacerbations in the propensity score-matched cohort (sensitivity analysis)

|  |  | Incidence rate  (95% CI) | Incidence rate ratio  (95% CI) | *P* value |
| --- | --- | --- | --- | --- |
| Model 1^*^ | Tobacco smoke only | 0.50 (0.28–0.72) | 1 |  |
|  | Biomass smoke only | 0.52 (0.28–0.76) | 1.05 (0.55–1.98) | 0.890 |
| Model 2^†^ | Tobacco smoke only | 0.48 (0.27–0.69) | 1 |  |
|  | Biomass smoke only | 0.54 (0.28–0.79) | 1.13 (0.59–2.16) | 0.721 |
| Model 3^‡^ | Tobacco smoke only | 0.44 (0.24–0.65) | 1 |  |
|  | Biomass smoke only | 0.57 (0.28–0.86) | 1.29 (0.63–2.62) | 0.489 |

^*^Model 1: unadjusted.

^†^Model 2: adjusted for sex.

^‡^Model 3: adjusted for sex, and medication possession ratios of long-acting muscarinic antagonists, long-acting β-agonists, and inhaled corticosteroids during the follow-up period as continuous variables.
